# A Green Approach for Preparing High-Loaded Sepiolite/Polymer Biocomposites

**DOI:** 10.3390/nano9010046

**Published:** 2018-12-31

**Authors:** Barbara Di Credico, Irene Tagliaro, Elkid Cobani, Lucia Conzatti, Massimiliano D’Arienzo, Luca Giannini, Simone Mascotto, Roberto Scotti, Paola Stagnaro, Luciano Tadiello

**Affiliations:** 1 Department of Materials Science, INSTM, University of Milano-Bicocca, Via R. Cozzi, 55, 20125 Milano, Italy; i.tagliaro@campus.unimib.it (I.T.); e.cobani@campus.unimib.it (E.C.); massimiliano.darienzo@unimib.it (M.D.); roberto.scotti@unimib.it (R.S.); 2 Istituto per lo Studio delle Macromolecole, ISMAC, CNR, 16149 Genova, Italy; lucia.conzatti@ge.ismac.cnr.it (L.C.); paola.stagnaro@ge.ismac.cnr.it (P.S.); 3 Pirelli Tyre SpA, 20126 Milano, Italy; luca.giannini@pirelli.com (L.G.); luciano.tadiello@pirelli.com (L.T.); 4 Institut für Anorganische und Angewandte Chemie, Universität Hamburg, 20146 Hamburg, Germany; simone.mascotto@chemie.uni-hamburg.de

**Keywords:** green composite, biocomposite, natural rubber latex, sepiolite, latex compounding technique, flocculation

## Abstract

Global industry is showing a great interest in the field of sustainability owing to the increased attention for ecological safety and utilization of renewable materials. For the scientific community, the challenge lies in the identification of greener synthetic approaches for reducing the environmental impact. In this context, we propose the preparation of novel biocomposites consisting of natural rubber latex (NRL) and sepiolite (Sep) fibers through the latex compounding technique (LCT), an ecofriendly approach where the filler is directly mixed with a stable elastomer colloid. This strategy favors a homogeneous dispersion of hydrophilic Sep fibers in the rubber matrix, allowing the production of high-loaded sepiolite/natural rubber (Sep/NR) without the use of surfactants. The main physicochemical parameters which control Sep aggregation processes in the aqueous medium were comprehensively investigated and a flocculation mechanism was proposed. The uniform Sep distribution in the rubber matrix, characteristic of the proposed LCT, and the percolative filler network improved the mechanical performances of Sep/NR biocomposites in comparison to those of analogous materials prepared by conventional melt-mixing. These outcomes indicate the suitability of the adopted sustainable procedure for the production of high-loaded clay–rubber nanocomposites with remarkable mechanical features.

## 1. Introduction

The demand for environmental process and products made from renewable and sustainable nonpetroleum-based resources is progressively growing in our society [1,2]. Among renewable materials, natural rubber latex (NRL) is the main elastomeric material obtained from the sap of the *Hevea Brasiliensis* tree, used for specific applications in different industry sectors, also combined with other rubbers and thermoplastics [3]. NRL appears as a milky colloid, based on poly(1,4-*cis*-isoprene), a highly unsaturated hydrocarbon polymer, and non-rubber components as water, carbohydrates, proteins, polypeptides, fatty acids, and phospholipids [4]. Natural rubber (NR) polymer has very high structural regularity, providing remarkable properties, i.e., good film-forming ability, high elasticity, low hysteresis and high resilience [5,6]. Within NRL, the nonrubber components contribute significantly to the excellent mechanical properties of NR [6]. Thus, NR is still indispensable for heavy-duty applications, especially tires [7,8] and rubber bearings for seismic, and is widely used to produce nanocomposites for protective thin films, adhesives, gloves, coatings, joints, pipes and tubes [9]. In addition, NRL can be easily employed for the preparation of rubber nanocomposites through the latex compounding technique (LCT). This method consists of mixing a stable aqueous elastomer dispersion (latex) with an aqueous dispersion of reinforcing filler. LCT offers several advantages: (i) absence of organic solvents; (ii) use of natural polymers available in latex form; (iii) since water acts as an excellent swelling and exfoliating agent, no expensive surface modification procedures for hydrophilic fillers, such as silica and clay, are required. Moreover, LCT represents an economic and ecofriendly alternative to the commonly employed melt mixing technique, which is based on the introduction of all the solid ingredients into a mixer [10]. In fact, during traditional melt mixing volatile organic compounds are commonly released, in contradiction with the increasing legislative restrictions about emissions.

Thus, LCT appears a promising and sustainable approach with clear benefits for the environment and the workers. In particular, LCT seems particularly appropriate for fillers like clays (e.g., talc, mica or phyllosilicates), whose efficient dispersion through melt mixing still remains a challenging issue [11], due to their poor compatibility with the organic matrix [12]. Clays are considered promising candidates for the strengthening of various types of polymers due to their lamellar structure with particles of high aspect ratio (AR) [13]. Besides, they are naturally occurring and readily available, almost ubiquitously, in large quantities compared to traditional silica and carbon black.

Among clays, sepiolite (Sep) is an abundant, natural and nontoxic phyllosilicate. Sep is a hydrated magnesium clay with unit cell formula Si_12_O_30_Mg_8_(OH, F)_4_(H_2_O)_4_•8H_2_O [14]. Structurally it is formed by fibrous particles of 0.5–10 μm length, constituted by alternating rods and tunnel-like cavities which extend throughout the fiber direction. Thanks to its natural abundance and low cost, Sep has been extensively used as filler in various polymeric matrices [15,16] to enhance their thermal, mechanical and barrier properties. However, the pristine bundle-like form of the Sep fibers inhibits its uniform dispersion within the polymeric matrix and their chemical modification is usually required to ensure interfacial interaction with polymers [17,18].

To date, there are only a limited number of studies regarding on clay/NR nanocomposites obtained by straightforward LCT [19,20,21,22]. In these reports, nanocomposites are generated from a coagulation process, activated by electrolytes as suitable coagulating agents. However, these compounds are usually nonbiodegradable, and may compromise also the properties and performances of the final materials. Moreover, most of the papers in literature do not investigate in depth the physicochemical parameters which rule the NR-filler interactions in aqueous media (e.g., pH, ξ-potential, size and shape of colloidal systems), determining the particle assembly in the polymer matrix and, consequently, the properties of bulk NR-based nanocomposites.

Herein, we propose an alternative, facile, and low-cost LCT approach to prepare ecofriendly biocomposites by incorporating Sep fibers into NRL matrix, through the flocculation (i.e., particle aggregation resulting from the bridging of several particles of a polymer) of a Sep/rubber mixed aqueous system in the absence of any organic solvent, surfactant or flocculating agent.

In particular, Sep fibers having a different surface charge were selected: pristine Sep, extracted from the Spanish landfill (SepS9) and a commercial Sep organically modified with a benzyl ammonium salt (SepB5). The fibers were added to NRL with a Sep/NRL weight ratio = 1:1, which allowed to obtain homogeneous Sep/NR biocomposites with a high filler concentration in quantitative yields. This process is more sustainable than melt mixing, in which the incorporation of very large amounts of filler requires significant energy input and may lead to the release of powder in the working environment, rising problems of compounding, dispersibility and contamination.

The main physicochemical parameters which control aggregation processes in the aqueous medium, i.e., pH, ξ-potential, size and shape of colloidal systems, and the morphological features of the final biocomposites were comprehensively investigated in order to figure out the interactions among Sep fibers and NR, and to propose a flocculation mechanism characteristic of each Sep/NR system. Finally, the mechanical properties of NR/Sep biocomposites obtained by LCT were determined by strain sweep analysis and compared to those of analogues composites prepared by conventional melt blending.

## 2. Materials and Methods

### 2.1. Materials

Sep Pangel S9 (SepS9) and the organically modified Sep Pangel B5 (SepB5, organically-modified with *N*,*N*-didodecyl-*N*-methyl-benzylammonium) were supplied by Tolsa and extracted from the landfill of Vallecas (Spain), LUDOX^®^ TM-50 colloidal silica 50 wt. % suspension in H_2_O by Sigma Aldrich (Sigma Aldrich, St. Louis, MO, USA). *N*-(1,3-Dimethylbutyl)-*N*′-phenyl-p-phenylenediamine (6PPD) was Santoflex-6PPD from Flexsys (Solutia Inc., Saint Louis, MO, USA). NRL Medium Ammonia (MA), (60% *w*/*w*) and poly(1,4-*cis*-isoprene) STR20 (NR) by Von Bundit (Von Bundit CO., Phuket, Thailand). HCl (37% *w*/*w*) and NH_4_OH (28–30% *w*/*w*) were purchased from Sigma-Aldrich. Milli-Q water with a resistivity 18.2 MΩ cm was utilized.

### 2.2. Preparation of Sep/NR Biocomposites by LCT

**Figure 1:**
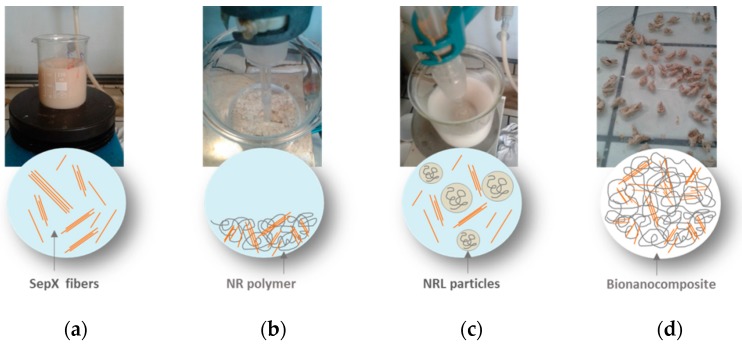
Scheme of the sepiolite/natural rubber (SepX/NR, with X = S9, B5) biocomposites preparation: (**a**) Sep dispersion in water; (**b**) mixing of the Sep aqueous dispersion with natural rubber latex (NRL); (**c**) flocculated SepX/NR biocomposite; and (**d**) dried SepX/NR biocomposite pieces.

Figure 1 shows the main steps of the preparation of the Sep/NR biocomposites by LCT. Twenty grams of SepX (X = S9 or B5) was dispersed in water (400 mL) with a mechanic stirrer (Velp Scientifica, Usmate (MB), Italy) at 200 rpm (right per minute). The mixing time was 60 min for SepS9 and 20 min for SepB5, corresponding to the optimal colloid relative dispersion needed to the next flocculation process (the optimal viscosity was identified by testing different mixing time). Subsequently, SepX dispersion was sonicated for 10 min and mixed again for 10 min. In another vessel, 33 g of NRL were diluted with 100 mL of distilled water and stirred for 10 min at 100 rpm with a mechanic stirrer. Successively, 0.2 g of finely ground 6PPD antioxidant were added to the solution. Then, the previous SepX dispersion was poured into the diluted NRL solution, under stirring at 500 rpm until the flocculation of the Sep/NR biocomposites was completed (30 s). The SepX/NR composite was separated from the liquid phase through filtration, and washed with water to remove ammonium residuals, portioned in small pieces and dried in a vacuum oven (pressure: 10 mbar) at 80 °C for 18 h. The obtained SepX/NR biocomposites were further homogenized in a two-roll mill at room temperature for 5 min. In order to highlight the role of the particle anisotropy, the same procedure was also applied to produce nanocomposites enclosing LUDOX^®^ TM-50 colloidal silica nanospheres.

Composites based on NR and SepX fillers were also prepared as reference materials by traditional melt mixing technique in a Brabender Plasti-Corder lab station internal mixer (65 mL mixing chamber, 0.6 filling factor): 25 g of NR and 25 g of SepX fibers were mixed together for 5 min at 90 °C and 60 rpm rotor speed. The obtained composite materials are called REF-(SepX/NR).

### 2.3. Morphological and Chemical Characterization of Colloidal Dispersion of SepX and NRL

To assess the hydrodynamic diameter of NRL particles, dynamic light scattering (DLS) was performed by a Malvern Zetasizer Nano Series ZS90 instrument (Malvern Panalytical Ltd., Malvern, UK). (Figure S1 in Supporting Information, SI). The NRL sample was diluted approximately 1:1 with Milli-Q water.

Morphology of SepX samples (Figure S2 of SI) was investigated by Scanning Electron Microscopy (SEM) analysis, performed by a Vega TS5136 XM Tescan microscope (Tescan Brno s.r.o., Kohoutovice, Czech Republic). The electron beam excitation was 30 kV at a beam current of 25 pA, and a working distance of 12 mm. In this configuration, the beam spot was 38 nm sized. Prior to SEM analysis, samples were gold-sputtered. ImageJ processing program (Image Processing and Analysis in Java, Version No 2, National Institutes of Health, Bethesda, MD, USA) was utilized to measure manually the Sep fibers dimensions.

The ζ-potential of the NRL particles was determined with Malvern Zetasizer Nano Series ZS90 in order to investigate the colloids stability. The NRL sample was diluted with deionized water until the pH value was approximately that of Milli-Q water (pH = 6.5).

The ζ-potential measurement on pristine NRL was performed at 25 °C at different pH by adding a suitable amount of HCl or NH_4_OH solution to NRL, maintaining the concentration at a constant level. ζ-potential measurements were carried out also on 100 mgL^−1^ SepX dispersions. The concentration of the suspension was the same as in the procedure of the preparation of Sep/NR biocomposite (see Paragraph 2.2). In detail, SepB5 aqueous dispersion was stirred for 10 min and sonicated for other 10 min, while SepS9 was stirred at different times (10 min, 40 min, 3 h, 24 h, Figure S3 of SI) before sonication. In such conditions, the SepS9 suspension after 10 min reached a pH = 8, which remains almost constant reaching the equilibrium (pH = 8.41) after 24 h. In addition, the ζ-potential measurements of SepX suspensions were performed at different pH by adding HCl or NH_4_OH at 25 °C.

The magnitude of the adsorption/ion exchange of ammonium ions on SepS9 was studied by using the Nessler method [23]. 10 g of SepS9 were dispersed in water (200 mL) under mechanic stirrer at 200 rpm for 60 min, successively sonicated for 10 min and mixed again for 10 min. In a second vessel, 0.1 g of dried NH_4_Cl (drying at 150 °C for 2 h) was dissolved in 50 mL of distilled water. The resultant NH_4_Cl solution was added to the SepS9 dispersion and stirred at a constant temperature (25 ± 2 °C). The pH of all solutions was adjusted by adding HCl 37% until pH was in the 6–8 range.

The concentration of the ammonium ions, in the aqueous phase containing SepS9, was analysed using the standard Nessler reagent method, employing a Varian Cary 4000 Spectrophotometer (Varian Inc., Palo Alto, CA, USA) under the following conditions: 300–600 nm wavelength range, 600 nm min^−1^ scan rate, 0.1 s time response, and 2 nm spectral band. 20 mL of sample aliquots were withdrawn at intervals of 3, 15, 30, 60, 120 min and separated by centrifugation. Then, the ammonium content in 0.1 mL of supernatant was analysed after adding 2.5 mL of distilled water and potassium sodium tartrate tetrahydrate to avoid the magnesium and calcium interference with the Nessler reagent. The corresponding kinetic data for SepS9 are depicted in Figure S4.

SepS9 dispersion was also investigated by Inductively Coupled Plasma Atomic Emission Spectrometry (ICP-AES), using a PerkinElmer OPTIMA7000 DV spectrophotometer (Perkin Elmer, Waltham, MA, USA), in order to investigate the potential release of metal ions. A dispersion of 50 g L^−1^ of SepS9 in Milli-Q water was mixed for 30 min and sonicated for 10 min. Then, the dispersion was filtered and the supernatant centrifuged for 30 min at 9000 rpm to eliminate any residual of SepS9 nanoparticles. The amount of Mg^2+^ in the supernatant was analyzed by ICP-AES (Table S1).

The experiments for the determination of Mg^2+^ through Capillary Electrophoresis were performed using a P/ACE MDQ Capillary Electrophoresis System (Beckman, Fullerton, CA, USA) equipped with UV detector. Fused-silica capillaries (75 μm I.D., 50 cm effective length, Beckman) were used. The analyses were performed by using indirect UV detection at wavelength of 214 nm. Electrophoresis separations were performed in a running buffer constituted of 6 mM HIBA, 5 mM 18-crown-6 ether and 5 mM imidazole adjusted to pH 4.5 with acetic acid. Analyses were performed by applying a constant voltage of 500 V cm^−1^ at 25 °C with a resulting current of about 18 μA. The separations took place at 25 kV during 6 min. The solutions were injected at the anodic end of the capillary for 10 s at 0.5 psi. Before injection, sample were diluted 1:5 (*v*/*v*) with water containing BaCl_2_ (IS) at a concentration of 40 μg mL^−1^. The amount of Mg^2+^ released at different time of mixing of SepS9 was reported in Table S1.

The first stages of the flocculation process in SepX/NR systems, were studied by a Jeol 3010 high-resolution transmission electron microscope (HR-TEM) (Mccrone Group, Westmont, IL, USA) operating at 300 kV with a high-resolution pole piece (0.17 nm point to point resolution) and equipped with a Gatan slow-scan 794 CCD camera. To visualize SepX/NRL interactions, 10 μL of the Sep/NRL mixture were taken from the flocculation system with the same concentration used in the Sep/NR composite preparation. Then, 1 mL aliquot of the flocculating dispersion was diluted 1:100, deposited on a carbon coated TEM grid, to monitor the evolution of the flocculation process.

### 2.4. Thermal, Spectroscopic and Morphological Characterization of SepX/NR Biocomposites

The thermogravimetric analysis (TGA) of SepX/NR biocomposite was performed with a Mettler Toledo TGA/DSC1 Star-e System. The variation of weight was analyzed by heating from 30 to 1000 °C, at 10 °C min^−1^, under an air flux of 50 mL min^−1^.

The crystalline structure of Sep fibers inside the SepX/NR biocomposites was confirmed by X-ray Diffraction (XRD), recorded in X’Pert Pro PXRD (Panalytical) diffractometer (Malvern Panalytical Ltd., Malvern, UK) in Bragg-Brentano geometry, using Cu K-α source with 1.54 Å wavelength.

The filler–rubber chemical interactions in SepX/NR biocomposites were analyzed through attenuated total reflection9—fourier transform infrared spectroscopy (ATR-FTIR) by a Perkin Elmer Spectrum 100 instrument (Perkin Elmer, Waltham, MA, USA) (4 cm^−1^ resolution, 650–4000 cm^−1^ range, 8 scans).

To assess the Sep distribution in Sep/NR biocomposites, SEM-energy dispersive spectroscopy (EDS) analysis was performed by A Vega TS5136 XM Tescan microscope (Tescan Brno s.r.o., Kohoutovice, Czech Republic) operating at 30 kV (25 pA beam current, 12 mm working distance, 38 nm beam spot). Samples were deposited onto a carbon adhesive disc and carbon-sputtered. A map of principal elements of Sep, such as Si, Al and Mg, was detected to visualize the filler dispersion within the rubber matrix.

The morphological investigation of composites was carried out by TEM using a Zeiss EM 900 microscope (Zeiss, Oberkochen, Germany) operating at 80 kV. Ultrathin sections (about 50 nm thick) of SepX/NR biocomposites previously embedded in an epoxy resin (70 °C, 24 h) were obtained with a Leica EM FCS cryo-ultramicrotome, equipped with a diamond knife, by keeping the samples at −130 °C.

Small-angle X-ray scattering (SAXS) was performed on beamline ID2 at the European Synchrotron Radiation Facility (ESRF, Grenoble FR) at a wavelength of 1 Å with a sample to detector distance of 2.5 m, yielding a total q-range from 0.001 to 0.15 Å^−1^. The scattering cross section per unit sample volume dΣ/dΩ (in cm^−1^) (scattered intensity I(q)) was obtained by using standard procedures including background subtraction and calibration given by ESRF. The Debye approximation [24] and the Guinier approach were adopted for the analysis of the NR and biocomposite curves, respectively. The scattering intensity I(q) in the Debye model is given by the following equation:

(1) $$I\left(q\right){=I}_{0}\frac{1}{{{\left[1+(q\xi \right)}^{2}]}^{2}}$$

where ξ is the correlation length of the scattering bodies, in this case associated with the crystalline domains. As particulate systems, the scattering of the Sep rods in the NR can be analyzed using the Guinier approach [25] to get information on the radius of the inorganic fibers and therefore on their dispersion degree. For one dimensional systems the scattering in the intermediate region of the curve can be approximated to:

(2) $$I\left(q\right)=\frac{I_{0}}{q}\exp\left(-\frac{q^{2}R_{g}{}^{2}}{2}\right)$$

(3) $$R_{g}{}^{2}{=R}^{2}/2$$

where R_g_ and R are the radius of gyration and the radius of the fiber, respectively.

These parameters are obtained from the linearity dependency of the so called Guinier plot $\ln\left(\mathrm{qI}\right)vs.{\;q}^{2}$.

### 2.5. Rheological and Mechanical Characterization of SepX/NR Biocomposites

Strain sweep experiments were carried out from 0.1 to 60% of strain at 100°C and 10 Hz with a Rubber Process Analyzer (RPA2000, Alpha Technologies, Bellingham, WA, USA). Both SepX/NR biocomposites and REF-(SepX/NR) samples were analyzed.

## 3. Results and Discussion

**Scheme 1:**
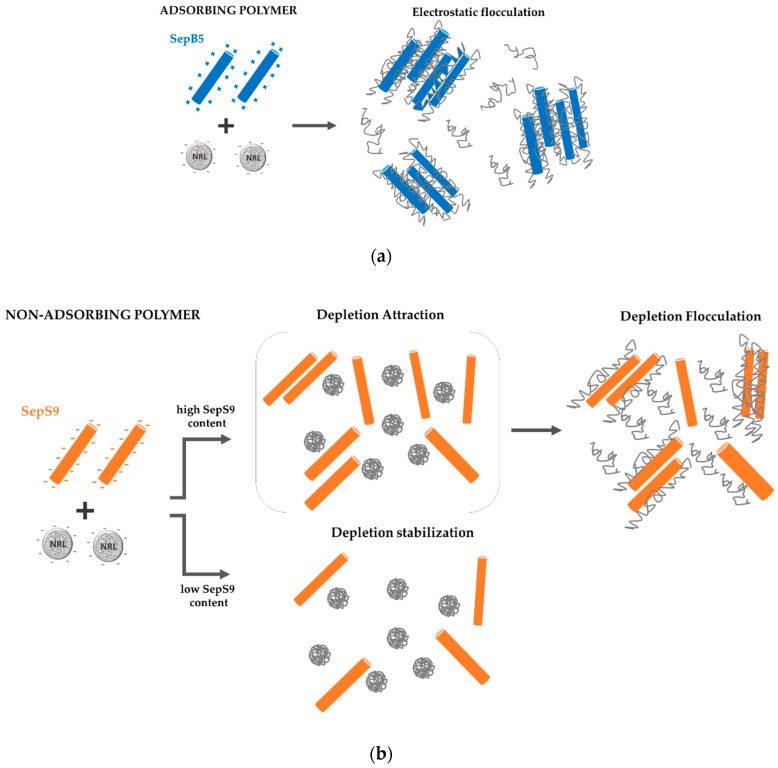
Proposed mechanisms for the flocculation process in (**a**) SepB5/NR and (**b**) SepS9/NR systems.

Sep/NR biocomposites were prepared through LCT, pouring an aqueous colloidal dispersion of SepX fibers into the NRL suspension under stirring, at different nominal SepX/NR weight ratios (Scheme 1) and without the presence of any surfactant or flocculating agent. Flocculation, i.e., a destabilization of the colloidal suspensions by bridging the particles by means of polymer chains and the consequent formation of a coagulum, rapidly occurs regardless the kind of Sep fibers employed. In particular, when high Sep filler loading (50 wt. %) was utilized, the binary colloidal dispersion flocculates in a few minutes, becoming an elastic coagulum. Generally, the knowledge on the nanomaterials dispersion by applying LCT suggests the use of low filler quantities in order to achieve a homogeneous coagulum [11,26]. Notwithstanding, the approach used here allowed to obtain high loaded Sep/NR biocomposites, envisaging the peculiar ability Sep fibers to spontaneously flocculate by interacting with NRL.

To elucidate the mechanism of this process, a preliminary characterization of the pristine NRL and Sep fibers was accomplished. Then, SepX/NR biocomposites were fully characterized and a flocculation mechanism, characteristic of each Sep/NR system, was finally proposed.

### 3.1. Characterization of SepX and NRL Colloidal Dispersions

Results of DLS analysis on NRL evidences a bimodal distribution of the hydrodynamic radius, indicating the presence of a fraction of particles with an average size of ~300 ± 130 nm (65%), and a second population (35%) with an average size of ~1200 ± 500 nm (Figure S1 of SI) [27]. Generally, NRL particles display high polydispersity with diameters comprised between 0.2 and 1 μm, due to their natural metabolic origin [27].

As already reported in our previous study [22], SEM micrographs on SepX fibers revealed that both SepS9 and SepB5 appear as rod like particles with similar AR strongly interconnected in bundles-like aggregates (Figure S2 in SI). Table 1 summarizes the average dimensions and AR of SepX fibers.

**Figure 2:**
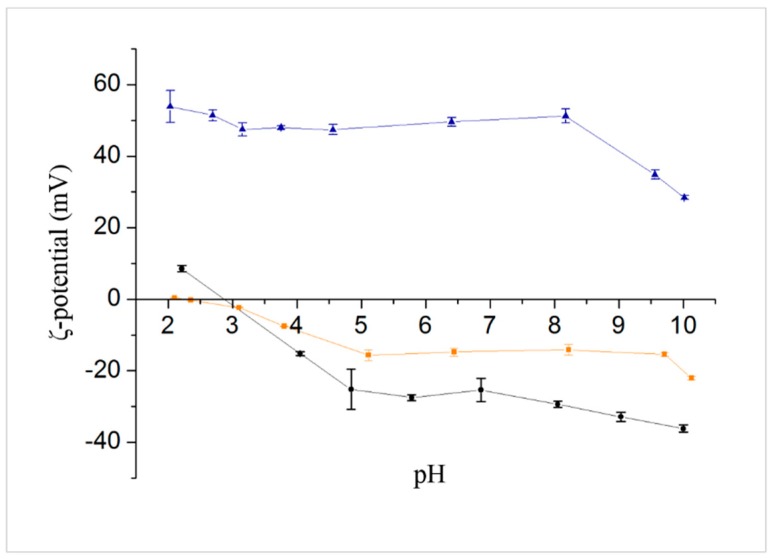
Trend of ζ-potential vs. pH for NRL–medium ammonia (MA) (●, - black line), SepS9 (■, - orange line) and SepB5 (▲, - blue line).

ζ-Potential analysis was performed on pristine NRL and SepX dispersion to define the charge of the electric double layer of these colloidal systems. In general, relatively high absolute values of ζ (±30 mV) indicate high colloidal stability. In the opposite case (ζ values in the range of ±5 mV), short-range attractive forces may exceed repulsion and coagulation/flocculation may easily occur. In an aqueous medium, the most common surface charge-determining ions for colloidal systems are H^+^ and OH^−^. Hence, the pH value readily affects the surface charge and, consequently, ζ-potential value. In particular, the ζ-potential becomes more positive or negative in magnitude with acidic and basic pH, respectively. Therefore, monitoring ζ values, at different pH, allows to determine the isoelectric point (IEP), i.e., the pH at which ζ-potential becomes zero and, consequently, colloids lose stability and agglomerate/flocculate (Figure 2).

The ζ-potential of pristine NRL-MA at pH 7 is −28 ± 0.05 mV. The negative surface charge is most likely provided by proteins and phospholipids amino and carboxyl functional groups, which can be easily ionized and whose charge balance give rise to a negative electric double layer [28]. Accordingly, decreasing the pH ζ of NRL-MA increases (●, - black line in Figure 2), reaching the IEP at pH ~ 2.9, in agreement with the literature [29].

As concerns on the Sep suspension, Sep bundles, once dispersed in the aqueous medium, form an arrangement of randomly intermeshed elongated fibers, kept together by physical interactions and hydrogen bonding. Water can easily penetrate through these structures, thus increasing the viscosity of the suspension, providing to the colloidal solution a pseudoplastic behaviour [30]. The optimal mixing time to obtain a SepX suspension was ~60 min for SepS9 and ~20 min for SepB5.

At pH 6.5, ζ value of the resulting colloidal systems was −34.8 ± 0.05 mV for SepS9, while for SepB5 modified with an alkylbenzyl ammonium salt, a positive ζ-potential was measured (+26.9 ± 0.05 mV).

SepS9 suspension (■, - orange line in Figure 2) has slightly positive ζ values (+0.38 at pH 2) at low equilibrium pH while negative ones at high equilibrium pH [31]. The IEP lies at pH ~ 2.4. As reported by Alkan et al. [31,32], at acidic pH SepS9 fibers in water solution display a positive ζ-potential due to the protonation of the surface groups, which undergo dehydroxylation or deprotonation at higher pH, leading to significant decrease of the ζ value. SepB5 (▲, - blue line in Figure 2) suspension shows instead a positive ζ-potential in the whole pH range, probably ascribable to the presence of alkylammonium salt as functionalizing group at the filler surface.

In order to evaluate all electro-kinetic and adsorption properties of SepX colloidal systems, further characterizations have been performed and reported in Paragraph 3 of SI.

### 3.2. Thermal, Spectroscopic and Morphological Characterization of Sep/NR Biocomposites

The loading of SepX fibers in the biocomposites was evaluated by TGA analysis (Figure S5, SI). In detail, SepX/NR biocomposites display a slight mass loss (∼1%) at 100 °C attributed to physisorbed water, while a major weight loss is detectable from 300 to 500 °C, due to thermal degradation of the rubber components and to the Sep organo-modifiers, when present [33]. The total amount of Sep fibers in the biocomposites thus matches with the remaining weight percentages at the end of the thermal evolution and corresponds to 45 and 50 wt. % for SepS9 and SepB5, respectively.

The XRD patterns of SepX/NR (Figure S6 of SI) clearly show the diffraction peaks ascribable to the SepX fibers, confirming that the flocculation process do not alter the Sep crystalline structure [34].

**Figure 3:**
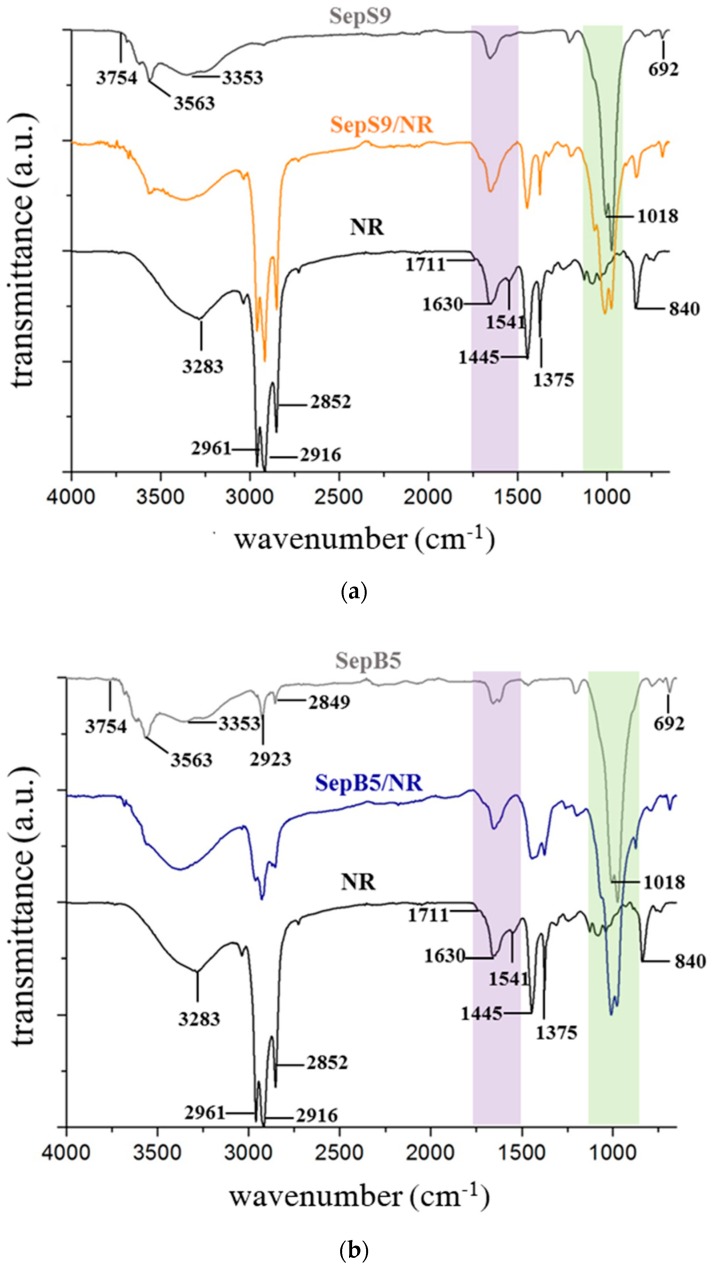
Fourier transform infrared spectroscopy (FTIR) spectra of (**a**) NR (black line), SepS9 (gray line), SepS9/NR biocomposite (orange line), (**b**) NR (black line), SepB5 (gray line), SepB5/NR biocomposite (blue line). The light green and violet shadow areas highlight the most significant spectral regions.

To investigate the nature of the potential interactions between Sep surfaces and NR matrix, ATR-FTIR spectra of SepX/NR biocomposites were collected and compared to those of NR, SepS9 and SepB5 (Figure 3).

The NR spectrum (black line in Figure 3) shows, as expected, the bands in the 3100–2850 cm^−1^ region due to the stretching of C–H bonds (both unsatured and satured) and those in the 1600–1350 cm^−1^ range, attributed to the stretching of C–C bonds and bending of C–H bonds [32]. At 840 cm^−1^, the C–H out-of-plane bending typical of the poly(1,4-*cis*-isoprene) chains is also detectable. Other bands can be attributed to some non-isoprene compounds, such as such as proteins and phospholipids [35], typically contained in the NR:NH groups of amines (3283 cm^−1^), carbonyl groups of esters (1748–1738 cm^−1^), carboxylic acids (1711 cm^−1^), and amides (1630 and 1541 cm^−1^).

SepS9 spectrum (gray line in Figure 3a) evidences the adsorption bands at: (i) 3700–3555 cm^−1^, attributable to the stretching vibrations of OH groups and water molecules bounded to octahedral magnesium centers, and (ii) 692 cm^−1^ the Mg–O–H bending vibration [36]. The band at 3754  cm^−1^ can be assigned to the silanol hydroxyl groups, while the typical Si–O bands of Sep tetrahedral sheets are present at 1057 and 1018 cm^−1^.

SepB5 spectrum (gray line in Figure 3b) presents, in addition to the typical SepS9 absorption bands [37], the vibrations of benzyl alkyl chains at 2923, 2849, 1661 cm^–1^, due to organic modifier.

Compared to SepS9 and SepB5 spectra, the stretching vibration of Si–O–Si and hydroxyl of Sep in both SepX/NR biocomposites resulted slightly shifted to higher wavenumbers (selected green region in Figure 3a,b), suggesting a weak interfacial interaction occurring between Sep and NR [38]. This effect may be connected to the physical adsorption of polymer polar or non-polar components present in NR onto Sep surface [39]. Moreover, the intensity of amide stretching bands at 1541 cm^−1^ were found to decrease in SepX/NR biocomposites (selected violet region in Figure 3a,b), envisaging the occurrence of intermolecular hydrogen bonds between protein molecules in NR and silanol groups of SepX [40].

These findings seem to support an interaction between non-rubber components in NR, such as lipids and proteins, and silanol functional groups on Sep surface, [40] able to promote the dispersion of the clays in the polymer matrix.

A deeper investigation of both filler/polymer interactions and morphological features of Sep/NR biocomposites was achieved by exploiting a multi-technique SEM, TEM and SAXS approach.

SEM micrographs (Figure S5 of SI) revealed that SepX/NR biocomposites are homogeneous materials, without any phase separation. In particular, a uniform distribution of Sep fibers through the whole rubber matrix and the absence of fiber agglomerates were assessed by Si elemental mapping.

The possible interactions occurring between SepX fibers and NRL particles were inspected by following the flocculation process with TEM.

**Figure 4:**
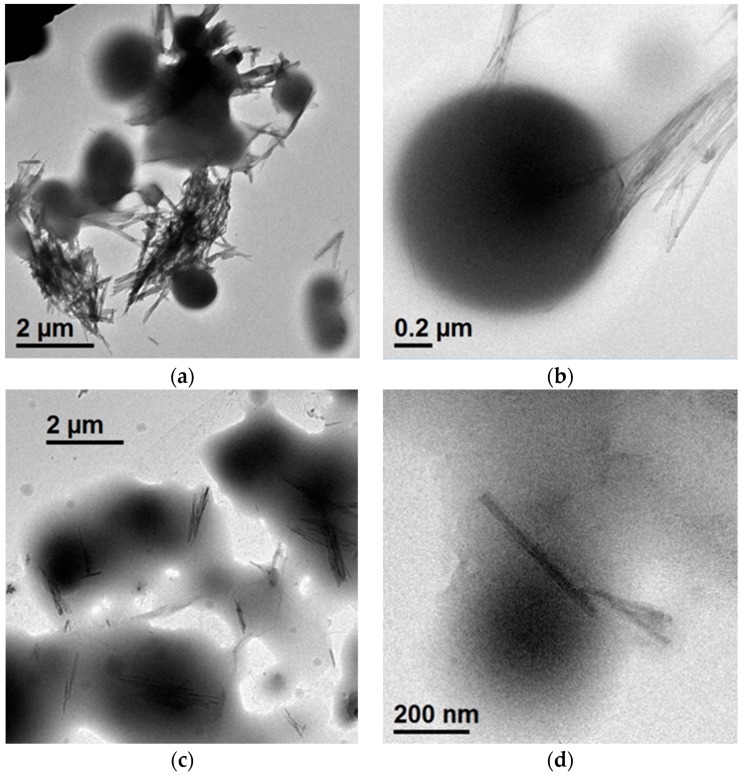
Transmission electron microscopy (TEM) images at different magnifications of dilute dispersions of SepX in NRL allowed to dry on a carbon-coated TEM grid: (**a**,**b**) SepS9/NR and (**c**,**d**) SepB5/NR colloidal systems.

TEM images of SepX/NR colloidal systems collected during the initial steps of mixing, i.e., before the flocculation starting, are reported in Figure 4.

In the case of SepS9/NR sample, filler aggregates lie among or in the periphery of the rubber micelles, indicating poor surface-mediated interactions between SepS9 and NRL (Figure 4a,b). Instead SepB5/NR appears constituted by homo-aggregated spherical NRL micelles [41] embedding some Sep fibers, some of which organized in bundles (Figure 4c,d). This suggests the existence of weak attractive interactions between SepB5 and NRL (short-distance forces), which may favour the adsorption of polymer chains onto Sep surfaces, thus supporting FTIR findings.

**Figure 5:**
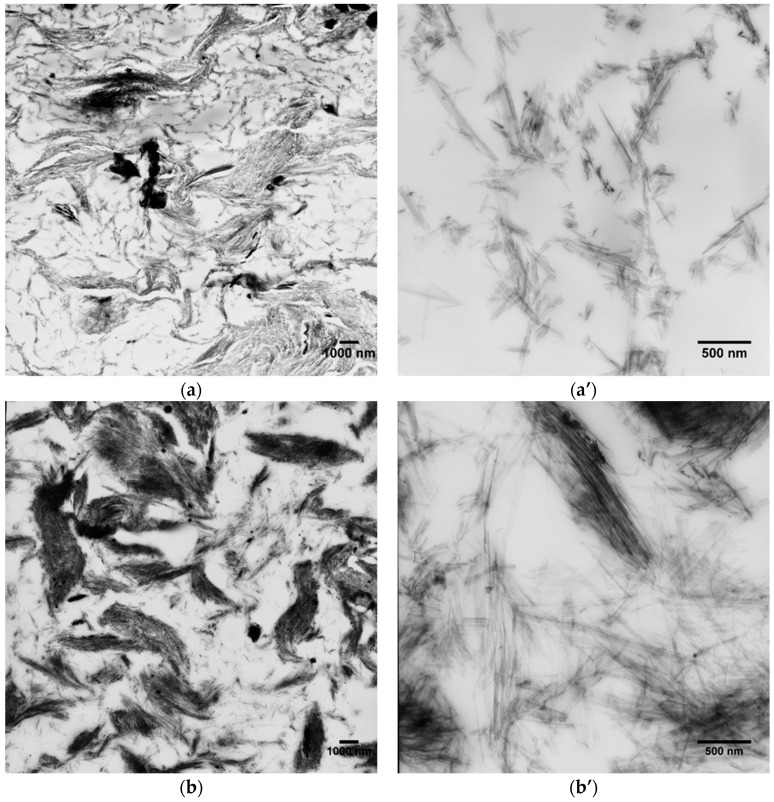
TEM images at different magnifications of (**a**,**a’**) SepS9/NR and (**b**,**b’**) SepB5/NR biocomposites.

TEM images collected on SepX/NR biocomposites, i.e., after the flocculation process, are shown in Figure 5.

For both SepX/NR biocomposites, the presence of an extended filler network structures, distributed through the whole material volume is observable, suggesting that percolation occurs (Figure 5a,b). The network appears constituted by large domains composed of self-assembled and partially oriented Sep fibers. This alignment can be attributed to large interface interaction of the anisotropic Sep fibers, forming differently oriented domains of rods preferentially aligned along the main axis. In particular, SepS9/NR display poorly compact filler aggregates with cross-section of ~100 nm, in which Sep fibers appear separated by small NR regions (Figure 5a’). Conversely, SepB5 domains are larger (cross-section ~400 nm) and constituted by highly interconnected Sep particles bridged by thin rubber layers (Figure 5b’). These results seem to confirm the occurrence of weak attractive interactions between SepB5 and NRL, which appear instead less pronounced in the SepS9/NR system.

In summary, TEM investigation reveals that the high SepX content guarantees for both SepX/NR biocomposites the formation of a percolative network, as demonstrated by the absence of wide regions constituted by pure NR. Hence, improved mechanical properties are expected (vide infra).

**Figure 6:**
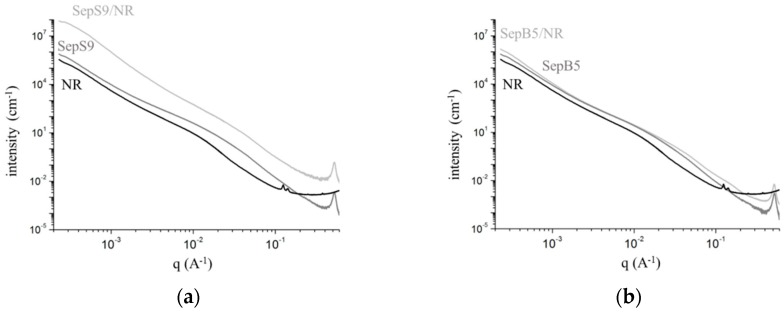
Scattering curves of (**a**) NR (black line), SepS9 (dark gray), SepS9/NR biocomposite (light gray), (**b**) NR (black line), SepB5 (dark gray), SepB5/NR biocomposite (light gray).

The SepX/NR biocomposites morphology was studied also by SAXS, in order to investigate more in depth the nanostructure of NR, SepX, and their composite assembly. Scattering curves obtained are shown in Figure 6. In the SAXS pattern of NR (Figure 6a) at high scattering vectors (q > 10^−1^ Å^−1^) the typical reflections of the orthorhombic unit cell of NR are observed [42]. As already proposed by Karino and coworkers [43], the remaining part of the curve could be described combining the scattering from aggregates [44] with the Debye approximation [45]. The shoulder centered at q values around 10^−2^ Å^−1^ indicates the presence of scattering bodies of 19 nm, most probably ascribable to the crystalline domains in the rubber, whereas at lower q the power law decay of −3.8 refers to interparticle scattering originated by micrometer sized particles with rough surface. The SAXS curves of the SepX fillers look both very similar (dark gray lines in Figure 6a,b). The strong peak at q = 0.5 Å^−1^ is related to the channel structure of the fillers [46]. The scattering of the Sep fibers can be analyzed by means of the Guinier approach (Figure S8 and Table S2) assuming a rod-like form factor.

In principle, this approach allows the determination of both radius and length of the scattering bodies. However, the latter parameter can only be assessed if the forward scattering at very low q vectors is visible, i.e., the system is high diluted. Therefore, since the Sep fibers form here bundle-like aggregates (steep power law at q < 0.002 Å^−1^), only the radii of the fibers, from the intermediate part of the curve, can be determined. These values are 11.5 nm and 11 nm for B5 and S9 samples, respectively, in good agreement with the SEM and TEM analyses.

Regarding the biocomposite samples (light gray lines), the peak at q = 0.5 Å^−1^ indicates that the filler is incorporated in the SepX/NR material. With respect to the curves of the single systems, the patterns show a different course. This effect is particularly visible for the SepS9/NR biocomposite at small scattering vectors. The higher scattering intensity is directly related with an enhancement of the interface area between the SepS9 fibers, thus suggesting that these inorganic fillers are much more homogeneously distributed in the polymer than SepB5, in accordance with the TEM results. In addition, the signals at *approx.* q = 10^−1^ Å^−1^ in the NR curve, proper of the crystalline domain of the rubber, disappear in the SepS9 biocomposite system, but not completely in the SepB5 one. This suggests that, once in contact with the SepS9 fibers, the polymer chains unfold much better generating a more homogeneous material, further showing that little interactions are established between organic and inorganic components.

The SAXS analysis supports the TEM investigation, showing the formation of different structural arrangement depending on the type of filler used.

### 3.3. Proposed Flocculation Mechanisms

Based on the gathered results and taking into account the most common reported flocculation mechanisms, i.e., charge neutralization, polymer bridging and electrostatic patch, two distinct possible flocculation pathways for SepS9/NR and SepB5/NR systems have been proposed (Scheme 1).

In the case of SepB5/NR (Scheme 1a), a mechanism simply based on charges neutralization can be inferred. In fact, at the working pH range (9–10), ζ-potential of SepB5 and NRL displays an opposite sign, suggesting the occurrence of an attractive potential among the filler and rubber particles, which can collide due to their Brownian motion [47,48]. The progressive adsorption of polymer segments on SepB5 surface, lowers the free energy of interaction among the fibers, decreasing their distance and leading to flocculation. It must be observed that the proposed mechanism is active only at high SepB5 loading; otherwise a charge reversal may occur resulting in the re-dispersion of the fibers [49].

These considerations are in agreement with the presence, at the early stages of mixing, of homo-aggregated NRL micelles embedding some SepB5 fibers (Figure 4c,d) while, in the final SepB5/NR biocomposite, of large domains constituted by aligned Sep fibers bridged by thin rubber layers (Figure 5b’).

In SepS9/NR, both the colloidal systems possess a negative surface charge at the working pH (see Figure 2). Thus, flocculation cannot be explained simply referring to the ζ-potential measurements. In fact, when a significant repulsive contribution to the inter-particle potential is present, experimental conditions other than surface charge should affect the flocculation process.

In the case of SepS9/NR, flocculation was observed only in the presence of the high loading of Sep fibers, while did not occur for more diluted colloid systems. This suggests that high filler content and a relatively low fraction of NR play a key role in promoting flocculation.

It is well known that attractions between colloidal particles can be conveniently induced by the addition of non-adsorbing polymers, due to the so-called depletion attraction [50,51]. The driving force of this phenomenon lies in the existence of a “depletion zone”, generated by the conformational entropic restrictions of the polymer coils which are not compensated by adsorption energy. According to these considerations, we can infer that in SepS9/NR system the filler loading is so high that NR is pushed out from the region among the fibers, leading to a higher concentration of the polymer in the colloidal solution (Scheme 1b, top). The difference in the osmotic pressure between these two regions results in the solvent flow out from the gap between Sep particles, inducing attractive forces which drive the flocculation. The hypothesis is supported by the fact that, at low SepS9 content flocculation does not take places, since the binary colloidal systems results sterically stabilized (Scheme 1b, bottom).

These observations are in accordance with TEM investigation on Sep9/NR which, at the early stages of mixing, shows (Figure 4a,b) that Sep bundles lies among or in the periphery of the rubber micelles indicating their poor interactions with NRL while, after flocculation reveals (Figure 5a,a’) the presence of poorly compact filler aggregates in the biocomposite. Moreover, SAXS analysis also supports the occurrence of weak interactions between the organic and inorganic components in Sep9/NR colloidal system, suggesting that, once in contact with the SepS9 fibers, the polymer chains unfold generating a homogeneous material.

Finally, it has to be mentioned that the efficacy of depletion forces is generally enhanced in the presence of colloidal particles with high AR, because they provide extensive and effective confinement regions [52]. This seems to indicate a remarkable influence of Sep anisotropy on the flocculation process of Sep9/NR system. To confirm this effect, a suspension of spherical silica nanoparticles, with surfaces properties similar to those of Sep fibers, was mixed with NRL under the same experimental conditions (i.e., filler/NR ratio, mixing time, stirring rate) used for the preparation of the biocomposites. As expected, a very slow and non-quantitative flocculation has been observed. These results strengthen the idea that high AR rods act as more efficient depletion agents and are suitable materials for the generation of soft matters [53,54].

In summary, the flocculation process in SepX/NR system can be ascribed to electrostatic or depletion attraction forces for SepB5 and SepS9, respectively. This is strongly connected both to the high filler content and to the peculiar anisotropic features of Sep fibers.

### 3.4. Dynamic-Mechanical Properties of SepX/NR Biocomposites

The dynamic-mechanical behaviour of SepX/NR biocomposites was compared to REF-SepX/NR composite, with the same filler content and obtained by conventional blending technique.

**Figure 7:**
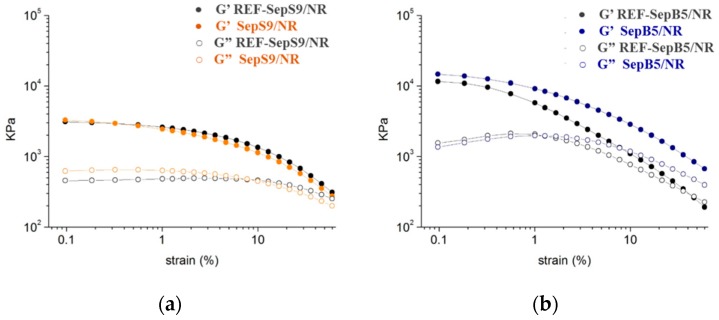
Storage modulus G’ (storage modulus, plain circle lines) and loss modulus G″ (loss modulus, empty circle lines) vs strain for (**a**) NR/SepS9 biocomposite (orange lines), REF-SepS9/NR (Reference-Sepiolite S9/Natural Rubber) nanocomposite (black lines) and for (**b**) NR/SepB5 biocomposite (blue lines), REF-SepB5/NR (black lines).

Strain sweep is a standard technique to quantify the contributions of the so-called “filler network” in composite materials formed by a percolating solid phase of filler particles embedded in a polymer matrix. Thus, in order to compare the behaviour of the filler network under large deformations or in the nonlinear region, strain sweep measurements at 80 °C were performed. The results of these measurements are shown in Figure 7 in terms of storage (G’) and loss moduli (G″), for both fillers SepS9 (Figure 7a) and SepB5 (Figure 7b).

In all uncured samples, results from shear measurements with strain amplitude increasing from 0.01 to 60% show a decrease of the storage modulus G’. This decrease in elastic modulus upon increasing the strain amplitude, i.e., Payne effect, is commonly related to the filler-filler interaction which are broken under strain. The short-distance forces between filler particles decrease strongly from low strain to high strain values. The breakdown of the filler network by increasing strain amplitude would also release the occluded rubber so that the effective filler volume fraction and hence the modulus decrease. For this reason, the Payne effect is used as a measure of filler networking originating from filler–filler interactions as well as from filler–polymer interactions, particularly for silicate-loaded rubber compounds.

In detail, the strain sweep curves obtained for SepS9/NR and REF-Sep9/NR samples are almost superimposable (Figure 7a) with the same G’ values both at low and high strain, advising that filler-filler and filler-rubber interactions are not affected by the mixing approach utilized.

G’ for SepB5/NR (Figure 7b) shows a significant but less pronounced sigmoidal decrease compared to REF-SepB5/NR and, consequently, a lower Payne effect. This suggests the formation of a more compact filler network in the biocomposite rather than in the blended one possibly due to better filler–rubber interactions [55].

In general, the low Payne effect observed for SepX/NR represents an indication of fast recovery of the filler network after a single large-amplitude deformation. As shown in TEM images, this may be related to the peculiar self-assembly and alignment of Sep fibers which improve their interfacial interaction with rubber.

G″ of nanocomposites is commonly related to the energy dissipation during deformation, which is associated with the breakage and rebuilding of filler-filler network and slippage of the polymer chains. Thus G″, known also as viscous modulus, describes the viscous behaviour of a nanocomposite. In the present case, the energy dissipation processes are quite similar for both SepX/NR and the related reference materials.

Similar to the storage modulus, G″ decreased at high strain amplitudes. However, biocomposites enclosing SepX fibers show only a moderate decay of G″. This effect may be attributed to the aforementioned Sep rubber interactions, which hinder the slippage of the macromolecular chains.

In addition, the maximum peak in G″ curves is slightly broaden and shifted to the higher strain amplitude for SepX/NR biocomposites in comparison with reference materials, indicating an improvement of the filler network stability during the strain sweep [56].

## 4. Conclusions

SepX/NR biocomposites were prepared by a novel green LCT approach, consisting of blending an aqueous colloidal suspension of SepX fibers in a NRL suspension, resulting in a fast flocculation process, without using any synthetic flocculation agent. The proposed method promotes a homogeneous dispersion of low hydrophilic Sep fibers in the rubber matrix, allowing the production of high-loaded Sep rubber composites.

TEM investigation performed at the early stages of mixing revealed that, when SepB5 fibers bearing alkylammonium salt functionalities are utilized, homo-aggregated NRL micelles embedding some SepB5 fibers are detectable while, in Sep9/NR colloidal system, Sep bundles lay among or in the periphery of the rubber micelles, thus suggesting their poor interactions with NRL. The same survey performed on the final biocomposite, revealed for SepB5/NR the presence of large and compact filler domains constituted by self-assembled and aligned Sep fibers interconnected by rubber. Conversely, for SepS9/NR poorly tight filler aggregates were detected in the biocomposite, indicating the occurrence of weak interactions between the organic and inorganic components.

SAXS analysis supported these outcomes pointing out that, in NR colloidal system, the polymer chains, once in contact with the SepS9 fibers, unfold leading to the formation of a homogeneous material. These results allowed us to propose a flocculation mechanism characteristic for both SepB5/NR and SepS9/NRL systems, based on electrostatic and depletion attraction forces, respectively, and remarkably connected both to the high content (50 wt. %) and to the peculiar anisotropy of Sep fibers.

Finally, the uniform Sep distribution in the rubber matrix, characteristic of the proposed LCT approach, and the percolative filler network formed give rise to enhanced mechanical properties for SepX/NR biocomposites in comparison to those of analogous materials prepared by conventional melt blending technique. Although this work focuses on Sep/NR nanocomposites, we expect that the developed sustainable synthetic strategy may be potentially extended to the production of high-loaded clay-rubber nanocomposites.

## Figures and Tables

**Table 1:** Dimensions * and AR of SepX fibers, obtained by scanning electron microscopy (SEM).

	Length (nm)	Cross-Section (nm)	AR
**SepB5**	1398 (±567)	67 (±15)	21 (±10)
**SepS9**	1781 (±1004)	80 (±21)	22 (±11)

* Calculated as average of the dimensions of 100 fibers in 20 different SEM micrographs.

## Supplementary Materials

The following are available online at http://www.mdpi.com/2079-4991/9/1/46/s1, Figure S1: DLS (dynamic light scattering) analysis of NRL (natural rubber latex), Figure S2: SEM micrographs of (a) SepS9 and (b), Figure S3: ζ-Potential of SepS9 after 20 min, 40 min, 3 h and 24 h of mixing SepB5, Figure S4: The weight percent of ammonium ions physi/chemisorbed onto SepS9, Table S1: Mg^2+^ amount released at different time of mixing of SepS9, Figure S5: TGA (thermogravimetric analysis) curves recorded under air flux of: (a) NR (continuous black line), SepS9 (grey dot line), SepS9/NR biocomposite (orange dashed line); and (b) NR (continuous black line), SepB5 (grey dot line), SepB5/NR biocomposite (blue dashed line), Figure S6: XRD (X-ray Diffraction) patterns of (a) SepS9 (black line) and the corresponding SepS9/NR biocomposite (orange line), and of (b) SepB5 (black line) and the corresponding SepB5/NR biocomposite (blue line), Figure S7: SEM (scanning electron microscopy) micrographs of (a) SepS9/NR biocomposite and (b) SepB5/NR biocomposite and the corresponding silicon-mapping performed by EDS on (a’) SepS9/NR biocomposite and (b’) SepB5/NR biocomposite, Figure S8:Guinier plot (circle lines) and liner fit (red line) of (a) SepS9 and (b) SepB5, Table S2. Parameters of the Guinier analysis of the SAXS curves of SepX.
