# Supplementary material for: A Green Approach for Preparing High-Loaded Sepiolite/Polymer Biocomposites
Source: Nanomaterials (Basel). 2018 Dec 31;9(1):46. doi: 10.3390/nano9010046 (PMC6359008; doi:10.3390/nano9010046)
Supplement: Supplementary file 1 [file nanomaterials-09-00046-s001.pdf]

nanomaterials

MDPI

Supporting Information

A green approach for preparing high-loaded clay/polymer bio-composites

Barbara Di Credico[1]*, Irene Tagliaro[1], Elkid Cobani[1], Lucia Conzatti[2], Massimiliano D'Arienzo[1], Luca Giannini[3], Simone Mascotto[4], Roberto Scotti[1], Paola Stagnaro[2], Luciano Tadiello[3]

[1] Department of Materials Science, INSTM, University of Milano-Bicocca, Via R. Cozzi, 55, 20125 Milano, Italy; i.tagliaro@campus.unimib.it (I.T.); e.cobani@campus.unimib.it (E.C.); massimiliano.darienzo@unimib.it (M.D.); roberto.scotti@unimib.it (R.S.)

[2] Istituto per lo Studio delle Macromolecole, ISMAC, CNR, Genova 16149, Italy; lucia.conzatti@ge.ismac.cnr.it (L.C.); paola.stagnaro@ge.ismac.cnr.it (P.S.)

[3] Pirelli Tyre SpA, Milano 20126, Italy; luca.giannini@pirelli.com(L.G.); luciano.tadiello@pirelli.com (L.T.)

[4] Institut für Anorganische und Angewandte Chemie, Universität Hamburg, Hamburg 20146, Germany; simone.mascotto@chemie.uni-hamburg.de (S.M.)

* Correspondence: barbara.dicredico@unimib.it (B.D.C.); Tel.: +39-02-64485189 (F.L.)

S1. DLS analysis of NRL

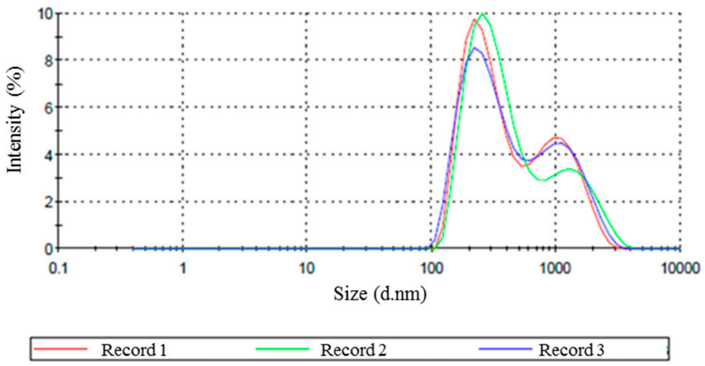

Figure S1. DLS (Dynamic Light Scattering) analysis of NRL (Natural Rubber Latex).

S2. Morphological analysis of Sep Fibers

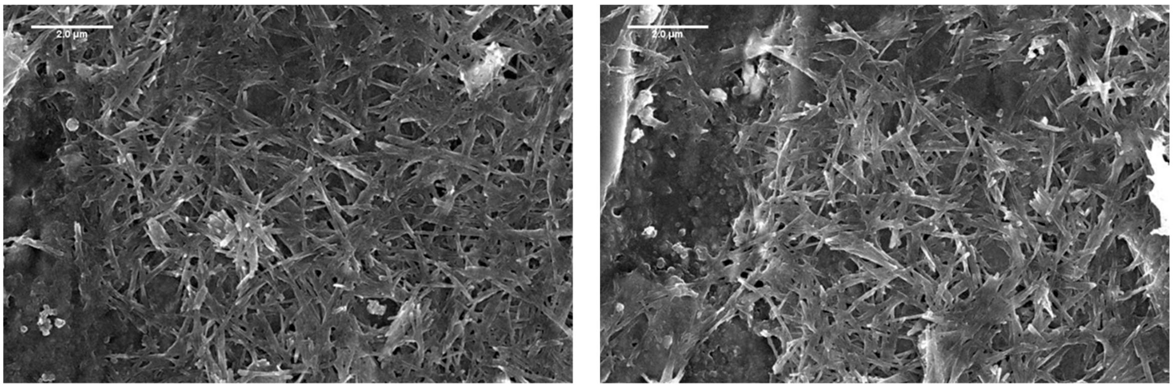

(**a**) (**b**)

Figure S2. SEM micrographs of (**a**) SepS9 and (**b**) SepB5.

S3. Electro-kinetic properties of colloidal systems

S3.1 ζ-Potential of SepS9

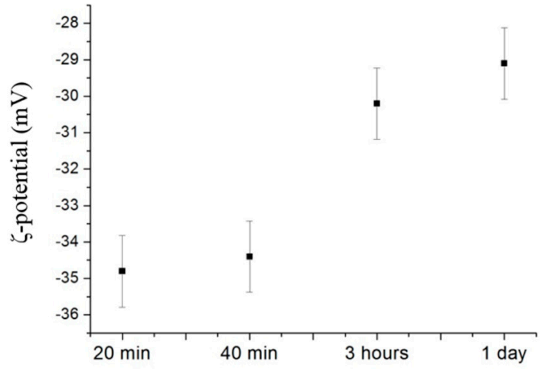

Figure S3. ζ-Potential of SepS9 after 20 min, 40 min, 3 h and 24 h of mixing.

A shift of the ζ-Potential to more positive values of about +5 mV is appreciable increasing the mixing time from 20 min to 3 h. This effect suggests a possible leach of Mg^{2+} ions, which may turn the ζ-potential of the fibers into more positive values.

S3.2 Adsorption/ion exchange of SepS9

The magnitude of the adsorption/ion exchange of SepS9 was studied in the presence of ammonium ions, which is in the NRL colloidal system. Adsorption of ammonium on the SepS9 was determined using the Nessler method. The corresponding kinetic data for SepS9 are depicted in Fig. S4. The results demonstrate that the 20% of the ions were absorbed during 120 min.

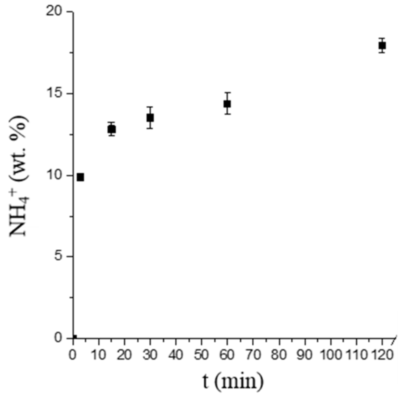

Figure S4. The weight percent of ammonium ions physi/chemisorbed onto SepS9.

Because ammonia solution in latex material inhibits bacteria action arising from high pH condition, hydrolysed fatty acid esters and in process form soaps that act as stabilizing bodies for the dispersed system, the ammonium adsorption disclosed in the presence of highly porous SepS9 perturbs the colloidal stability and could favour the flocculation process.

S3.3 SepS9 Magnesium leaching

The leaching of magnesium was evaluated during the mixing time by means of ICP-OES (Table S1). Capillary Electrophoresis (CE) analysis was performed in order to confirm the presence of magnesium cations, not coordinated in chemical molecules, such as MgOH.

Table S1. Mg^{2+} amount released at different time of mixing of SepS9.

	ICP-OES (mgL^{-1})	CE (mgL^{-1})
**20 min**	3.07	3.23
**1 day**	4.69	4.13

A small amount of magnesium ions was detected, that could affect the ζ-potential suggesting the influence of the magnesium ions on the flocculation.

S4. Analysis of bio-composites

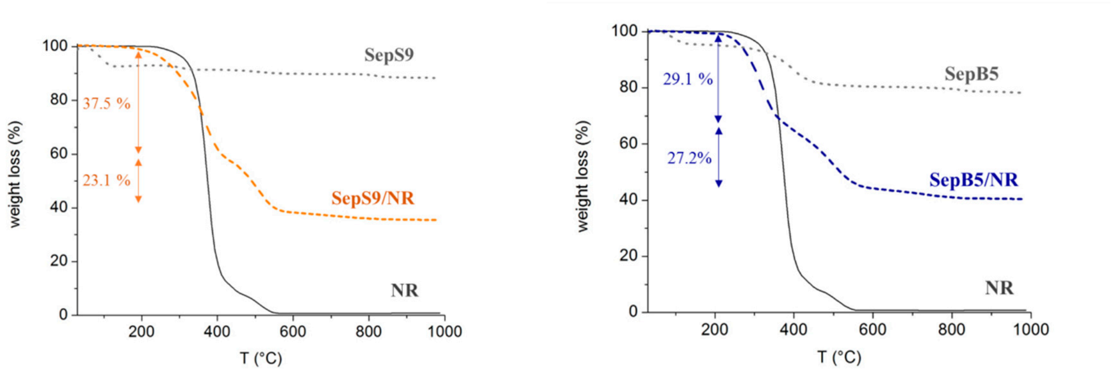

(**a**) (**b**)

Figure S5. TGA (Thermogravimetric Analysis) curves recorded under air flux of: (**a**) NR (continuous black line), SepS9 (grey dot line), SepS9/NR bio-composite (orange dashed line); and (**b**) NR (continuous black line), SepB5 (grey dot line), SepB5/NR bio-composite (blue dashed line).

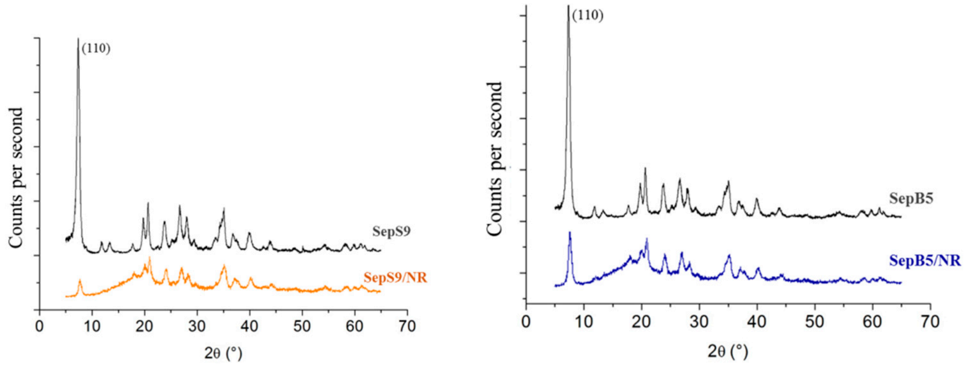

(**a**) (**b**)

Figure S6. XRD (X-Ray Diffraction) patterns of (**a**) SepS9 (black line) and the corresponding SepS9/NR bio-composite (orange line), and of (**b**) SepB5 (black line) and the corresponding SepB5/NR bio-composite (blue line).

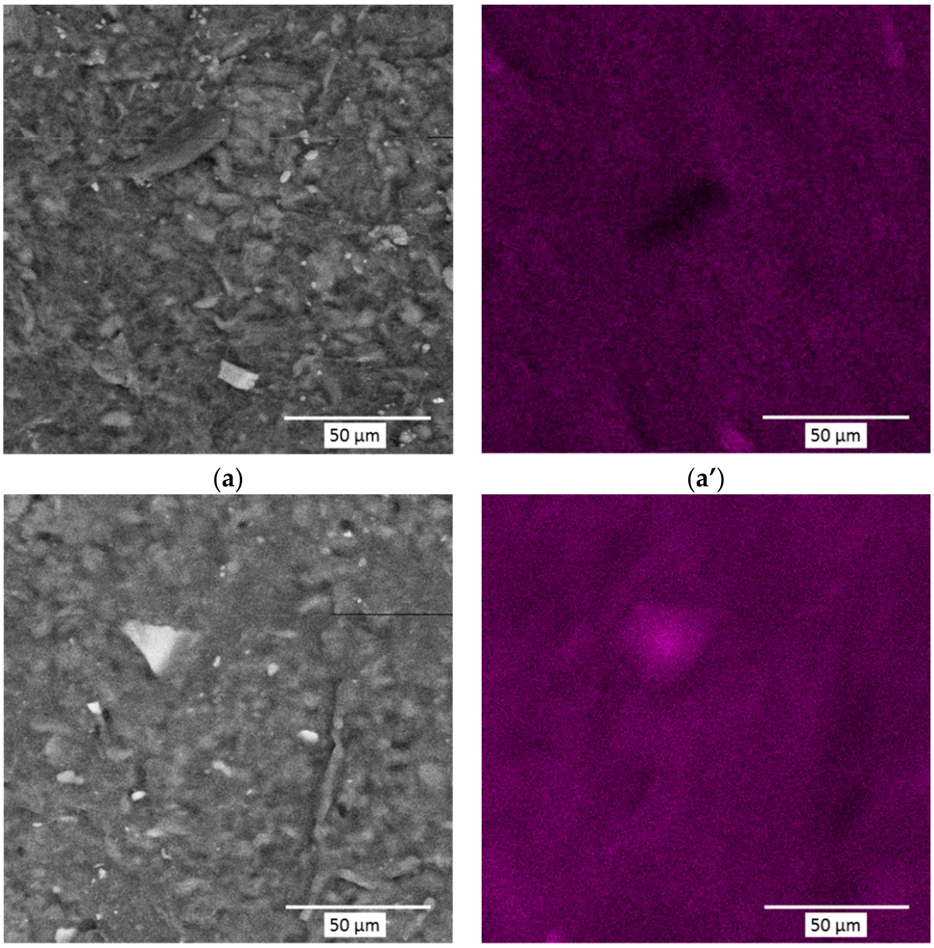

(**a**) (**a′**) (**b**) (**b′**)

Figure S7. SEM (Scanning Electron Microscopy) micrographs of (**a**) SepS9/NR bio-composite and (**b**) SepB5/NR bio-composite and the corresponding silicon-mapping performed by EDS on (**a′**) SepS9/NR bio-composite and (**b′**) SepB5/NR bio-composite.

S5. Guinier approach for SAXS analysis

The scattering of the clay particles can be analyzed by means of the Guinier approach assuming a rod-like form factor [1].

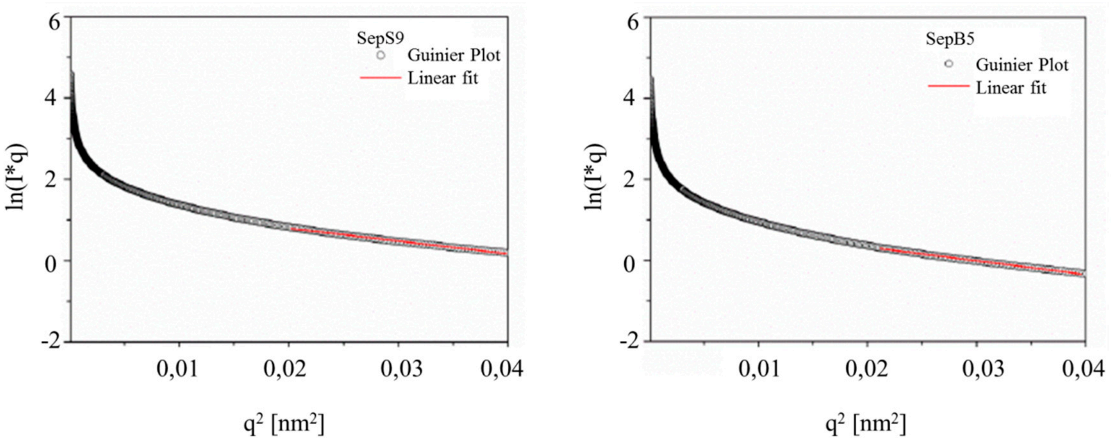

(**a**) (**b**)

Figure S8. Guinier plot (circle lines) and liner fit (red line) of (**a**) SepS9 and (**b**) SepB5.

Table S2. Parameters of the Guinier analysis of the SAXS curves of SepX.

	Slope (nm)	Rg (nm)	R (nm)
SepS9	31	7.9	11
SepB5	33	8.1	11.5

References

1. Dékány, I.; Turi, L.; Fonseca, A.; Nagy, J.B. The structure of acid treated sepiolites: small-angle X-ray scattering and multi MAS-NMR investigations. *Appl. Clay Sci.* **1999**, *14*, 141–160. doi:https://doi.org/10.1016/S0169-1317(98)00056-8

© 2018 by the authors. Submitted for possible open access publication under the terms and conditions of the Creative Commons Attribution (CC BY) license (http://creativecommons.org/licenses/by/4.0/).
